# Design and Psychometrics of the Assessment Instrument for Innovation Capabilities of Medical Sciences Universities Using the Cube Model Approach

**Published:** 2020-02

**Authors:** Mashallah TORABI, Reza SAFDARI, Saharnaz NEDJAT, Kazem MOHAMMAD, Maryam GOODARZI, Hossein DARGAHI

**Affiliations:** 1. Health Information Management Research Center, School of Allied Medical Sciences, Tehran University of Medical Sciences, Tehran, Iran; 2. Department of Epidemiology and Biostatistics, School of Public Health, Tehran University of Medical Sciences, Tehran, Iran; 3. Technology Management Department, Faculty of Management and Economy, Science and Research Branch, Islamic Azad University, Tehran, Iran; 4. Department of Health Care Management, School of Allied Medical Sciences, Tehran University of Medical Sciences, Tehran, Iran

**Keywords:** Instrument design, Psychometrics, Innovation, Assessment

## Abstract

**Background:**

To investigate any subject, a scholar needs a suitable instrument to collect the required information with the utmost accuracy and the least amount of error. Therefore, this study aimed at designing and conducting a psychometric analysis of an assessment instrument for innovation capabilities of Medical Sciences Universities using the Cube Model Approach.

**Methods:**

This study began by searching in questionnaires in the fields of input and process, considering innovation outcomes. Accordingly, a preliminary questionnaire was developed, and in the second stage, to determine the validity of the designed instrument, the face validity, content validity, and construct validity of the instrument were approved, and in the third stage, using Cronbach’s alpha, its reliability was assessed. At first 200 phrases were obtained, finally, 25 questions were initially approved in three areas of structure (input), innovation processes, and output. All the phrases were retained in the face validity and content validity carried out quantitatively and qualitatively.

**Results:**

Exploratory Factor Analysis was performed on 25 items, and finally the terms were set in six factors. These factors explained 53.19% of the total variance. The rotated factor loading for all questions was obtained more than 0.3, and therefore, no questions were eliminated. Calculation of the Cronbach’s alpha coefficient confirmed the high internal consistency of the questionnaire (0.762).

**Conclusion:**

This instrument was designed for the first time in the context of Iranian academic culture and seems to be a suitable instrument for the assessment of innovation capabilities, considering its adequate validity and reliability, simplicity, and practicality.

## Introduction

Developing an innovation program is a priority to move across knowledge boundaries and to appropriately concentrate on competitive advantage in organizations as well as scientific and business institutions. Therefore, designing an instrument is required to identify the status of innovation in each organization, group, or individual and to analyze the strengths and paths necessary to make possible the analysis of the strengths and finding suitable paths to mobilize resources (1). In the current competitive world, innovations in the business model are inevitable for the effective entry of knowledge-based organizations and institutions in international arena (2). Innovation is the process of utilizing mental abilities to create a new thought or concept and turning it into a useful and valuable product, service, or method of operation. Furthermore, to achieve sustainable growth, organizations must be innovative. In addition, measuring and controlling the set of ethical codes in innovative projects is quintessential (3–5). Therefore, all knowledge-based organizations, including universities, should always set the measurement of the innovation capability of their organization as their main activity in order to be powerful and have a significant competitive advantage. Innovation potential is considered as one of the competitive factors in that the development of innovative products plays an important role in competitive advantages (6).

In this regard, the mere attention to the number of innovations is not a reliable indicator for assessing the performance of innovation among different industries, and that in the leading institutions, efforts have been made to provide a more complete picture of the existing conditions of the organization by assessing the potential of innovation (7). Innovation is not a product of individual work but is a product of a set of influential factors including teamwork, process, and proper application of technologies. Most innovation measurement instruments have been designed by international institutions with the approach to assess the innovational capability of business and social institutions. Innovation indicators were proposed based on three indicators of process, input, and outcome to measure innovation capability at the university. Input indicators include the criteria related to the approach and strategy of the university, such as the university resource level dedicated to industry/university interaction, the amount of time allocated to entrepreneurship and innovation; process indicators include entrepreneurial culture and university innovation capability, such as the percentage of technology/engineering staff and students engaged in volunteer innovation and entrepreneurship activities, the number of university patents transmitted to industry partners at no cost, and output indicators including indicators related to the impact of the university on the ecosystem such as the number of start-ups/spinoffs and the percentage of graduates working in technology-related businesses (8). Innovation measurement models conducted in different countries of the world are based on either a detailed or holistic approach. However, the cubic or architectural approach addresses both approaches and defines the process of innovation and output in three structure parts (9).

To investigate any subject, a researcher needs a proper instrument to collect the required information with the utmost accuracy and the least amount of error (10). On the other hand, an instrument designed in a particular country reflects only the language and culture of that country, and if used elsewhere, even if there is an exact translation, there will be a bulk of problems due to the inappropriateness of content (11).

Thus, due to the importance of the issue and the lack of appropriate instruments for evaluation, a decision was made to conduct a study aimed at designing and psychometrically assessing innovation capability at Tehran University of Medical Sciences.

## Methods

In designing instruments and questionnaires, expressions can be designed through reviewing studies in the field of desired concept, observations and clinical interviews, qualitative researches such as grounded theory, choice of expressions of existing instruments, or the combination of all the above-mentioned methods (12, 13). The study began with a search of similar question-naires that could facilitate the desired activity. Taking into account the novelty of the research subject and the fact that similar research was not found based on the searches carried out elsewhere, it was attempted to collect questionnaires in the fields of input, process, and innovation results.

For this purpose, a preliminary search was carried out in authoritative databases inside and outside the country, library resources and research findings from organizations such as United Nations Educational, Scientific and Cultural Organization (UNESCO), European Innovation Scoreboard 2005 (14–21), in which the phrases of the initial questionnaire were designed with 200 questions. Subsequently, the expressions were evaluated by a group of experts using the Delphi method in two steps to ensure that they accurately assess what they claimed to measure. Finally, the final questionnaire was designed with 25 questions in three areas of structure (input), innovation processes, and output.

### Psychometric analysis of the instruments

In order to determine the validity of the designed instruments, face validity methods, content validity, and factor analysis of the structure were used.

### Face validity

Quantitative and qualitative approaches were used to determine the validity of the questionnaire. The qualitative aspects are conceptual and quantitative aspects are numerical (22). In this study, both quantitative and qualitative aspects of the questionnaire were examined by one of the participants. To assess the qualitative aspect of the questionnaire, 10 people (23) were selected among the directors and staff of Tehran University of Medical Sciences to examine its difficulty level 1 (the difficulty of understanding the phrases and words)|. After the correction of cases according to the participants’ views, in the next step, to reduce the phrases, omit the inappropriate phrases, and determine the importance of each of the phrases, the quantitative method regarding the effect of item 1 was used. Each phrase was scored using a 5-point Likert scale (from not important at all to completely important) and using the formula (Frequency (%) Importance), and if the impact score equaled or exceeded one and a half, the phrase was appropriate for the next analysis and was maintained (24).

### Content validity

To determine the content validity, qualitative and quantitative methods were used. Determining the content validity was based on the judgment of experienced experts in the field of designing instruments and experienced management in various innovation issues. In the qualitative study of the content, researchers asked 10 experts (23) to provide feedback after evaluating the quality of the questionnaire based on the following criteria: 1) observing the grammar, 2) the use of proper words, 3) necessity, importance, placement of phrases in their proper place, and 4) appropriate scoring. To quantitatively assess the validity of the content, two indicators of content validity ratio (CVR) and content validity index (CVI) were used. In order to determine the CVR, 10 experts from the above-mentioned areas were asked to examine each item based on a 3-part scale (necessary, useful but not necessary, not necessary). Lawshe Table was used to determine the minimum value of the index. If the number obtained from the mentioned table is more than 0.62 (based on the assessment of 10 experts), the presence of the related item with a significant statistical level (*P*<0.05) is necessary in this instrument (24). At the same time, in order to determine the relevance, simplicity, and clarity of each of the statements in the questionnaire, the experts were asked to examine the CVI, based on the Waltz and Bussel content validity index. These three criteria were examined independently by experts on a 4-point Likert scale for each item. If the CVI score is greater than 0.79, the statement in the question is appropriate; if the CVI score is 0.70 to 0.79, the statement is a questionable item, and if the score is less than 0.70, the item is unacceptable and should be deleted (25).

### Construct validity

The innovation capability instrument is designed for the first time, to confirm the content validity of the structure, exploratory factor analysis was used to explain the correlation patterns among the statements of each domain (26). In order to perform factor analysis, in terms of the number of subjects in various studies, 5–10 samples were considered sufficient for each item, and even some have considered sufficient three samples for each item (27). The number of statements examined was 25; therefore, a sample, 10 times of the number of statements of the questionnaires, was determined from among managers, the staff, and students. Given 10% lack of complete response and loss, finally, 200 samples were selected. Sampling was done as a census at a college of Tehran University of Medical Sciences.

### Reliability of the measurement instrument

In order to determine the reliability of the instrument, two methods of determining internal consistency and reliability were used. To measure internal consistency, Cronbach’s alpha (or alpha coefficient) was used. Cronbach’s Alpha represents the validity of a group of items that assess a structure. At this stage, using the random sampling method, the instrument was completed by 50 participants from the previous stage. To have good and adequate internal consistency, Cronbach’s alpha should be between 0.70 and 0.80 (28).

To observe ethical considerations, permission was granted from the Faculty to carry out the research. The participants were informed about the goals of the study, and informed consent was obtained from them. They were assured that their information would be confidential and anonymous and that they could exit from the study at any time.

The present study was approved by the Research Council of Tehran University of Medical Sciences dated 7 Sep 2015 and was approved under the project number 27189.

For data analysis, SPSS software (version 19, Chicago, IL, USA) was run and for the descriptive and inferential tests, mean, Cronbach’s alpha, and factor analysis were applied.

## Results

Overall, 219 individuals participated in the study including 174 students and 45 faculty members with the mean age of 27.51 ± 5.2 yr old. Table 1 shows the demographic information of the participants.

**Table 1:** Demographic characteristics of the units under study

***Demographic information***		***No. (percentage)***
Gender	Male	67(30.6)
	Female	152(69.4)
Age (yr)	Fewer than 20	28(12.8)
	20–29	120(54.8)
	30–39	36(16.4)
	40–49	26(11.9)
	50–60	9(4.1)
	mean	27.51±5.2
Academic members’ work experience	1–10	12(30.8)
	11–20	9(23)
	21–30	18(46.2)
	mean	10.08±8.7
	Single	13(6.8)
Marital Status	Married	140(73.7)
	Divorced	11(5.8)
	Dead	26(13.7)
	B.S.	111(63.8)
Student education	MA	43(24.7)
	Ph.D.	20(11.50)
Academic members’ education	MA	27(60)
	Ph.D.	18(40)

### Face validity

The values of instrument items ranged from 3.3 to 4.2, and given that these values were more than 1.5, all of them were considered important and appropriate for the target group and were maintained for subsequent stages.

### Content validity

Using the Lawshe table and based on the evaluation of 10 experts, if the CVR is more than 0.62, then the relevant item is essential and important. The CVR for all questions was between 0.63 and 0.8 and more than 0.62; hence, no questions were deleted in this section. Content Validity Index (CVI) was also obtained for all questions 0.76 to 1, and given that these values were more than 0.7, no question was deleted in this section. According to the results of CVI, mean relevance, mean clarity, mean simplicity, and S-CVI/Ave comprised 78.40±10.28, 70.40±10.98, 77.60±13.93, and 84.56±8.77, respectively.

### Construct validity

Exploratory factor analysis was performed on 25 items using principal components and Varimax rotation. The values of Bartlett’s Test and the Kaiser-Meyer-Olkin statistic indicate that the sample number is sufficient to perform the factor analysis. The values of Kaiser-Meyer-Olkin statistic should be more than 0.7. The value was equivalent to 0.72 in this study (Bartlett X2=663.56, df=300, *P*=0.000). The number of factors obtained based on 25 questions was six.

These six factors accounted for 53.19% of the total variance. The rotational load factor for all questions was more than 0.3, so no questions were deleted. These six factors were placed in three fields of results, process, and input after assessing the questions and considering the architecture approach. The first factor with seven questions based on the content of the questions was named continuous innovation; the second factor with four questions was named ordered innovation; the third factor with five questions was named the overactive innovation, the fourth factor fourth with four questions was named the active innovation; the fifth factor with four questions was named transformative innovation; the six factor with one question was named innovation resulting from the pressure of science or technology. Factor 6 and factor 1 were placed in the field of results; factors 2, 3, and 5 were placed in the input domain, and factor 4 was placed in the process field. In order to determine the reliability of the instrument, the internal consistency test was performed. The Cronbach’s alpha of all questions was obtained 0.80 (Table 2).

**Table 2:** Results of the exploratory factor analysis, the extracted factors, Eigen value, the predicted percentage of variance, and Cronbach’s alpha

***Rotated Component Matrix^a^***						
***Variable***	***Component***					
	***Factor 1***	***Factor 2***	***Factor 3***	***Factor 4***	***Factor 5***	***Factor 6***
q1		.735				
q2				0.445		
q3						.786
q4		.759				
q5				.684		
q6		.580				
q7	.435					
q8				.367		
q9		.425				
q10	.446					
q11					.325	
q12				.439		
q13	.737					
q14	.643					
q15	.678					
q16	.684					
q17					.330	
q18			.426			
q19			.673			
q20			.646			
q21			.340			
q22			.697			
q23	.312					
q24					.647	
q25					.421	
Eigen value	4.78	2.41	1.93	1.59	1.33	1.26
Cumulative predicted Percentage of the variance	13.56	11.68	8.86	7.15	5.97	5.97
Cronbach’s alpha	0.70	0.72	0.67	0.57	0.41	

## Discussion

The proposed instrument is a valid instrument in terms of the validity and reliability, simplicity and degree of applicability to evaluate the innovation potential in universities.

Until recently, measures of innovation have often been organized by government agencies, statistical offices, and academic institutions to meet their needs. Therefore, the results were completely different in importance and could not be easily compared (29), so it is necessary to provide a unified instrument for this purpose.

Several instruments were obtained, each with its own weaknesses and strengths, used for a particular issue of innovation. For example, a number of researchers in their studies used self-developed instruments with respect to their organization, which did not have adequate psychometric measures. For example, in a descriptive study conducted in Pakistan and in eastern cities of Africa, a researcher-made questionnaire was used to measure the innovation of teachers in teaching. This 29-item questionnaire evaluated faculty members’ opinions about knowledge and attitude towards innovation, their view of available resources and time, their view of organizational support, their motivation for innovation, and the challenges ahead. However, this questionnaire was not assessed psychometrically based on standard scientific rules (30). In a number of other studies, innovation has been assessed by instruments that emphasize the individual factor, and the role of organization as an influencing factor on this variable is neglected (31–33). The third group used the instrument of Amid et al. This instrument assesses innovation in organizations and at managerial levels. This questionnaire has 22 questions in five main areas of environmental innovation, leadership innovation, individual innovation, environment-feedback, and individual-feedback (34).

To determine the status of innovation, another group also used the Ekwall innovation status questionnaire, modified in later editions by Isaacson et al., called Modified Innovation Status Inventory (35). This questionnaire, using 10 factors, evaluates the organization’s innovation status and has been used since 1980 to identify the status of innovation in companies and business organizations in different countries (36, 37). The so-called instrument is intended to determine the status of innovation in organizations and bureaus and is never used in educational environments.

Besides all the so-called constraints, another important issue is that the existing instruments examine somehow innovation, but none of them measures the innovation capability. Regarding the definition of innovation, i.e. the process of utilizing mental abilities to create a new thought or concept and turning it into a useful product, service, or method of operation and a value creator procedure, the need to examine the power of innovation as an effective and important factor is of particular significance (38). This is one of the most important reasons for the need for an instrument in this regard.

In the design of innovation measurement models carried out in different countries of the world, the approaches adopted address the issue via either detailed or holistic approach. Although the process of evaluating innovation may not always be correct, the preparation and standardization of various tests is the first and most important step in creating and promoting innovation in organizations (39). Innovation is influenced by social and cultural conditions of each society and the lack of indigenous and standard instruments appropriate to the context and characteristics of Iranian universities, on the one hand, and the lack of any other study in this regard in Iran, designing an instrument is required with the combination of both detailed and holistic approach in three sections (9), along with other influential factors.

The proposed instrument was designed for the first time in Iran, and exploratory factor analysis was used to ensure the construct validity because of the newness of the instrument according to the experts’ views. Exploratory factor analysis was used for construct validity in some studies because of first designed a new instrument (40, 41). In this research, the Cronbach’s alpha coefficient indicates the internal consistency of the instrument expressions, and this confirms the reliability of the proposed instrument.

### Limitations

The lack of similarity of the instruments with the designed instrument, not in Iran, nor elsewhere with the so-called approach, prevents the possibility of comparing this instrument with others.

## Conclusion

This instrument was designed for the first time in the context of the academic culture of Iran and seems to be a suitable instrument for evaluating the innovation capability with regard to the appropriate reliability, validity, simplicity, and practicality.

## Ethical considerations

Ethical issues (Including plagiarism, informed consent, misconduct, data fabrication and/or falsification, double publication and/or submission, redundancy, etc.) have been completely observed by the authors.
